# The effects of donor age on organ transplants: A review and implications for aging research

**DOI:** 10.1016/j.exger.2018.06.019

**Published:** 2018-09

**Authors:** Jose Carlos Dayoub, Franco Cortese, Andreja Anžič, Tjaša Grum, João Pedro de Magalhães

**Affiliations:** a Integrative Genomics of Ageing Group, Institute of Ageing and Chronic Disease, University of Liverpool, William Henry Duncan Building, Room 281, 6 West Derby Street, Liverpool L7 8TX, United Kingdom; b Biogerontology Research Foundation, Research Department, Oxford, United Kingdom

**Keywords:** Biogerontology, Geroscience, Organ transplantation, Graft survival, Senescence

## Abstract

Despite the considerable amount of data available on the effect of donor age upon the outcomes of organ transplantation, these still represent an underutilized resource in aging research. In this review, we have compiled relevant studies that analyze the effect of donor age in graft and patient survival following liver, kidney, pancreas, heart, lung and cornea transplantation, with the aim of deriving insights into possible differential aging rates between the different organs. Overall, older donor age is associated with worse outcomes for all the organs studied. Nonetheless, the donor age from which the negative effects upon graft or patient survival starts to be significant varies between organs. In kidney transplantation, this age is within the third decade of life while the data for heart transplantation suggest a significant effect starting from donors over age 40. This threshold was less defined in liver transplantation where it ranges between 30 and 50 years. The results for the pancreas are also suggestive of a detrimental effect starting at a donor age of around 40, although these are mainly derived from simultaneous pancreas-kidney transplantation data. In lung transplantation, a clear effect was only seen for donors over 65, with negative effects of donor age upon transplantation outcomes likely beginning after age 50. Corneal transplants appear to be less affected by donor age as the majority of studies were unable to find any effect of donor age during the first few years posttransplantation. Overall, patterns of the effect of donor age in patient and graft survival were observed for several organ types and placed in the context of knowledge on aging.

## Highlights

• Data on the effects of donor age upon the outcomes of organ transplantation are an underutilized resource in biogerontology
• We compiled data on the effect of donor age following liver, kidney, pancreas, heart, lung and cornea transplantation
• Older donor age is associated with worse outcomes for all the organs studied
• The donor age from which the negative effects upon survival starts to be significant varies between organs

## 1 Introduction

The progressive deterioration and loss of functionality that characterizes the aging process affects different systems and organs of the body in different ways. Through measurements at different levels, numerous microscopic, macroscopic and functional age related changes have been robustly characterized in many different tissues and organs (Craig et al., 2015). In what represents a more indirect but holistic approach, organ transplantation data give researchers the opportunity to compare the outcomes elicited by grafts from donors of different ages in order to yield novel insights into the nature and pathogenesis of organ-intrinsic aging, the effect of aged organs upon the rate and pathogenesis of organismal aging in young hosts, and the effect of a young host environment upon the rate and pathophysiology of aging in organs from elderly donors. In general, the use of transplantation data to yield insights into the nature, rate and pathophysiology of both organ and organismal aging has remained an underutilized approach within the broader field of biogerontology, despite its potential to yield novel insights into the dynamics of organ-intrinsic and organismal aging.

Since the beginning of clinical organ transplantation more than five decades ago, the outcomes of different types of transplantation have been improving, especially with regard to short-term postoperative outcomes, and the use of older donors has become more and more frequent. In 2012, the number of adult transplants in the United Kingdom alone was 2881 for kidney, 246 for pancreas, 792 for liver, 136 for heart and 179 for lung. Indeed, in the same year 35% of the donors were 60 years old or over, compared to the 14% registered in 2003 (Johnson et al., 2014). Many clinical trials and retrospective studies using different databases have analyzed the effect that the age of the donor has upon postoperative outcomes for particular organs, sometimes with conflicting results (Alexander and Vaughn, 1991; Marino et al., 1995; Keith et al., 2004; Stehlik et al., 2012; Roig et al., 2015; Bittle et al., 2013; Wakefield et al., 2015). Here, we review the literature pertaining to the main abdominal and thoracic organs used for transplantation, as well as for the cornea, with the aim of providing a more comprehensive analysis of the extent with which donor age affects patient outcomes for each specific organ and tissue included in our analysis, and to infer possible differences in intra-organ and inter-organ rates of aging.

## 2 Results

Below we review the literature on the effects of donor age on the clinical outcomes after transplantation for several organs and the cornea, with a focus on graft and patient survivals.

### 2.1 Liver

Marino et al. (1995) found donor age to be an independent predictor of graft failure following liver transplantation in the first 90 days post operation, as well as during the 1.12 to 2.6 years of follow-up in their retrospective double-center study with 419 adults transplanted between 1992 and 1993. The stratification of donor age into <60 and ≥60 years old showed a significant reduction in graft survival at 23 months post operation in recipients with older donors, as well as a tendency toward the same direction when patient survival was measured. Further modeling using donor age as a continuous variable and adjusting for additional risk factors revealed no variations in the risk of graft failure for donors <45 years old. They found that the risk of graft failure began to increase progressively with the age of the donor for donors 45 years of age and older (Marino et al., 1995). Using a larger cohort and after stratifying the donors by age into <20, 20–29, 30–39, 40–49 and ≥50 years old, Detre et al. analyzed patient outcomes at 6-month following initial liver transplantation and found that, from donors aged 30–39, the risk of graft failure increased progressively with donor age. In the case of patient survival, the effect was not noticed until donors 40–49 years old, and did not increase further with the next age group (Detre et al., 1995). In another study with a median follow-up of three years, Feng et al. found the same progressive increase of the risk of graft failure with donor age, starting in the group of recipients with donors 40–49 years old as compared to those with donors aged <40 years. In this case, however, additional stratification of the reference group would have been needed to know if the effect starts at an earlier donor age in this population (Feng et al., 2006).

On the other hand, in their univariate analysis, after stratifying donors according to age at 10 year intervals Hoofnagle et al. were unable to find a significant decrease in graft survival at 3, 6, 12 and 24 months for donors <50 years of age. The multivariate analysis at 3 months, splitting donors into two groups with a cut-off age of 50, confirmed the detrimental effect of older donors on graft survival but found that this effect was largely restricted to those livers assessed by the harvesting surgeon as of poor or fair quality. When only livers that had been assessed as being of good quality were considered, the effect of donor age lost significance. Although no multivariate analysis was performed using longer follow-up times, the survival curves for graft failure showed that the first few months posttransplantation accounted for most of the difference seen in graft survival between old and young donors. Compared to graft survival, donor age showed a more moderate effect on patient survival (Hoofnagle et al., 1996). Burroughs et al. (2006) also analyzed patient survival using data of 34,664 first adult liver transplants from the European Liver Transplant Registry and found that donors 41–60 years of age had worse survival outcomes at 3 and 12 months than those aged ≤40, but better survival outcomes than those >60.

The impact of donor age is also noticeable in the long term histology of the transplanted liver. Rifai et al. found that older donor age was associated with the presence of ductopenia and higher fibrosis scores in the biopsies from patients who were alive 10 years posttransplantation. These authors determined that donor age <36 years old was a predictor of normal histology in the biopsies (Rifai et al., 2004). Overall, taking all these data into account together, liver donor age has a measurable impact on graft and recipient survival.

### 2.2 Kidney

In a study involving 50,322 patients of primary deceased donor kidney transplantation, Keith et al. found that the age of the donor was among the three main pretransplantation factors affecting long term patient survival, along with the age of the recipient and a renal diagnosis of diabetes. The stratification of donors into several age groups revealed that the 5- and 10-year patient survivals adjusted by different covariates started to decrease from donors aged 36 to 40 years. Furthermore, stratification of the recipients into <40, 41–54, and ≥55 year age groups revealed that, for each group, older donors were associated with lower survival curves at 10 years posttransplantation (Keith et al., 2004). Oppenheimer et al. studied the cases of 3365 recipients with a functioning graft at one year after kidney transplantation and observed a linear increase in the risk of long term graft failure and patient death with increasing donor age that started to become significant for donors 30–40 years old, as compared to the reference group of <20 years old. Their multivariate analysis also included the risk of acute rejection, which did not differ between the groups (Oppenheimer et al., 2004). Laging et al. analyzed the effect of donor age using data from living and deceased donors separately and observed that, in both cases, the risk of graft failure censored for death and uncensored graft failure started to increase exponentially from donors over 30 years old. Despite the risk curves for the two types of donors being quasiparallel, deceased donors conferred a greater risk of graft failure than living donors along all the age range studied. For deceased donor transplantation, an increased risk of graft failure was also found with pediatric donors (living donors of this age were absent) (Laging et al., 2012).

Other studies have analyzed the impact of donor age in the outcomes of kidney transplantation without providing additional information about the age at which this effect begins to be seen. Stratifying the donors into two groups with a cut-off age of 50, Moreso et al. found donor age to be an independent risk factor for graft failure for recipients who had not experienced any episodes of acute rejection. Further analysis revealed that donor age was an independent risk factor for graft failure due to chronic transplant nephropathy (Moreso et al., 1999). This condition, also known as chronic allograft nephropathy (CAN), is characterized by a progressive decline in renal function accompanied by histopathological changes affecting the glomerular, tubular, vascular and interstitial compartments, and is the main cause of late graft failure following renal transplantation (Birnbaum et al., 2009). Comparing recipients of donors 50–69 years old with those ≥70, Chavalitdhamrong et al. found transplants from older donors to increase the risk of overall graft failure, death-censored graft failure and patient death. Similar results were obtained when comparing donors 60–69 vs ≥70, but in this case the effect of donor age on death-censored graft failure was not significant (Chavalitdhamrong et al., 2008).

Along with donor age, the age of the recipient is one of the variables commonly found to affect the outcomes in organ transplantation. The interaction between donor and recipient age has been analyzed in different studies for kidney transplantation with conflicting results. In a secondary analysis of their data, Chavalitdhamrong et al. (2008) found that the kidneys from donors ≥70 years old conferred a higher risk of graft failure and patient death in recipients aged 41 to 60 than in those aged >60. Using data from 1269 patients, 44 of whom had a donor older than 55, Waiser et al. (2000) found that kidneys from donors >55 increased the risk of graft failure in young recipients (<55) almost two-fold but had no significant effect in older recipients (>55). In their univariate analysis of 201 live donor kidney transplantation recipients, Lee et al. (2014) found that the 10-year graft survival of recipients with donors >10 years younger than the recipients was reduced in comparison to those instances in which the ages of recipient and donor were matched (i.e. <10 years of difference between the recipient and their donor).

Also in the context of interactions between donor and recipient age, in a study with 40,289 cases of single renal transplantation, Meier-Kriesche et al. found donor and recipient age to have an additive detrimental effect on the risk of chronic allograft failure, for which the authors give a definition intended to exclude causes of graft failure not associated with CAN. This combined effect in graft survival became synergistic after the third year posttransplantation (Meier-Kriesche et al., 2002). In another large scale study involving recipients of deceased donor renal transplants, Kasiske and Snyder also studied the interaction between donor and recipient age after stratifying both groups into several age ranges. When the risk of graft failure in the different age combinations between recipients and donors was measured, no consistent deviation from the expected additive risk was found (Kasiske and Snyder, 2002). Independently of how the age match or mismatch between donors and recipients affects survival outcomes, the data for kidney transplantation clearly show that donor age has an impact in recipient and graft survival.

### 2.3 Pancreas

Due to the relatively high risk of surgical complications and a lower demand, the average age of donors accepted for pancreas transplantation is lower than for the other organs, resulting in a shortage of data on patient survival curves and risk of complications for pancreas transplants from old donors. This shortage of data is more marked for Pancreas Transplantation Alone (PTA) because overall only a small percentage of pancreas transplants are of this type. For this reason, in the present review we have also included studies that measure pancreas graft survival after simultaneous pancreas-kidney (SPK) and pancreas after kidney (PAK) transplantations.

Only a few studies have addressed the effect of donor age on the outcomes of PTA independently. In a retrospective analysis of all pancreas transplantations performed in the United State between 2000 and 2004, Gruessner and Sutherland found reduced graft survival in patients of PTA receiving grafts from donors ≥50 years old, as compared to those under 50, which they interpreted as being due to higher rates of technical failure (TF) (defined as early graft losses attributed to vascular thrombosis or removal because of bleeding, anastomotic leaks, pancreatitis, or infection) in recipients of pancreases from donors >50 years of age. When only TF-free patients were considered, the rates of graft survival between recipients of younger and older donors showed no significant differences. Similar results were found for patients of SPK transplantation (Gruessner and Sutherland, 2005). In a more recent study, the same database was analyzed for the period of 2006–2010. When donors were stratified by age into 0–14, 15–29, 30–44 and >44 age groups, no significant relationship between donor age and graft survival after PTA transplantation was observed. In patients of SPK, however, the relative risk of pancreas graft failure started to increase with donors aged 30–44 years (Gruessner, 2011). In a different study, Sutherland et al. were also unable to find a significant effect of donor age on graft survival and TF after PTA, and observed only a tendency toward a worse outcome in the case of SPK transplantation. However, as in the case of the two previous studies, this lack of significance may have been due to having an inadequate sample size (Sutherland et al., 2001).

In line with the results by Gruessner and Sutherland (2005), and using a cohort that included SPK, PAK and PTA patients, Schenker et al. (2008) found that the main reason for the reduced graft survival rates at 1, 5 and 10 years in recipients with older donors (≥45) was the higher rate of early graft failure due to technical complications in this group. The association between donor age and TF was further analyzed by Finger et al. in a retrospective study of deceased donor pancreas transplants performed in adults at the University of Minnesota between 1998 and 2011. They found donor age >50 to be an independent risk factor for graft loss within the first 90 days posttransplantation due to TF (Finger et al., 2013).

The detrimental effect of donor age upon long-term pancreas graft survival, independently of the early outcomes, has also been reported. Salvalaggio et al. (2007) considered early and late pancreas graft outcomes separately in SPK patients, and found donor age ≥45 to be an independent risk factor for graft failure at both 90 days and 5 years after transplantation. On the other hand, Kayler et al. (2013) found that donors aged ≥40 reduced the 3-year pancreas graft survival after SPK in both young recipients (<40) and old recipients (≥40). Therefore, while data for pancreas is more limited than for other organs, the above data nonetheless suggest that older donors are an important risk factor implicated in graft survival.

### 2.4 Heart

Different studies have found an effect of donor age in patient survival as well as in the risk of developing cardiac allograft vasculopathy (CAV), an accelerated form of coronary artery disease developed in the transplanted graft and one of the main causes of late mortality in heart transplant patients. The 29th report of the International Society of Heart and Lung Transplantation, based on transplants performed between 2001 and 2006 in 394 centers worldwide, concluded that increasing donor age was associated with higher rates of mortality at 1, 5 and 15 years posttransplantation, as well as with a higher risk for the development of CAV within 5 years posttransplantation (Stehlik et al., 2012). Stratifying the donors into those above and below 50 years of age, Roig et al. also found a higher incidence of CAV in recipients of transplants from older donors at 5 and 10 years (but not at 1 year) posttransplantation, which persisted after adjusting for different confounding factors. In contrast, the higher rate of global mortality in patients with older donors found in the multivariate analysis lost significance after adjustment for donor cause of death, donor smoking history, recipient age, and induction and cyclosporine therapy. With respect to acute mortality, defined by the authors as deaths up to one month posttransplantation, no differences between the two groups were found both with and without adjustment (Roig et al., 2015). Kuschmann et al. also considered donor cause of death, donor smoking history, recipient age and data on the induction and cyclosporine therapy in their 3 years follow-up study of 774 adult transplant recipients in Germany between 2006 and 2008. Of these variables, only donor cause of death (nontraumatic vs traumatic) and recipient age were associated with patient survival after heart transplantation in the multiple Cox regression analysis. This analysis also found a significant reduction in patient survival with increasing donor age (Kutschmann et al., 2014).

Stratifying donors into several age groups, other studies have obtained a more concrete idea of the effect of donor age upon medium and long-term patient survival following heart transplantation. In a 5-year follow-up retrospective study involving 22,960 adult transplant recipients between 2000 and 2012, Weber et al. found that after adjusting for different donor and recipient characteristics, heart transplants from donors 40–49 years of age were associated with a slightly increased risk of mortality with respect to those aged 18–39, and that this difference increased progressively in each successive age group (50–55 and >55) (Weber et al., 2014). This is consistent with 40–49 years of age marking the beginning of a curve of decreasing patient survival, but further stratification of the reference group would have been needed to determine whether the effect starts any earlier. A similar result was obtained by Gupta et al. who, using a cut-point analysis, concluded that transplants from donors aged 40 years or older were associated with poorer survival rates independently of the other risk factors found in their single-center study involving 667 recipients (Gupta et al., 2004). On the other hand, Nagji et al. analyzed the effect of different donor ages on the onset of CAV and found that, starting from donors 30–39 years old, the risk of developing this pathology increased progressively with the age of the donor. A further analysis comparing the effect of transplants from donors under 20 years old with those from donors aged 50 or over upon different recipient age groups (18–29.9, 30–39.9, 40–49.9 and ≥50 years old) revealed that transplants from older donors were associated with a higher risk of developing CAV in all the recipient age intervals considered (Nagji et al., 2010). As seen in other organs, the data presented here also show an evident detrimental effect of donor age in patient outcomes following heart transplantation.

### 2.5 Lung

Baldwin et al. studied the effect of donor age on graft survival using the records of 8860 cases of adult lung transplantation performed between 2005 and 2011, as provided by the American Organ Procurement and Transplantation Network (OPTN). When treating donor age as a continuous variable, a slight increase in the risk of graft failure at 1 year was observed with the use of donors younger than 18 years which, after declining to a minimum between donors aged 30 and 50 years, increased with donor age progressively throughout the rest of the age range studied. Further stratification of donor age gave similar results: recipients of donors <18 presented an increased risk of 1-year graft failure with respect to the reference group (18–29 years), but revealed no significant differences in this outcome for transplants from donors aged between 18 and 64. Donors ≥65 exerted a strong negative effect on graft survival, with a relative risk of graft failure at 1 year that reached more than two-fold that of transplant recipients from the reference group. This effect of the oldest donors, however, lost significance when it was measured at 5 years posttransplantation in recipients who had not experienced graft failure during the first year posttransplantation, 67 of whom had a donor 65 years old or over (Baldwin et al., 2013). Using the same database for the period of 2000–2009, Bittle et al. obtained similar results when analyzing the effect of donor age in patient survival. While the risk of mortality at 30 days did not differ significantly between the different age groups (18–34, 35–54, 55–64 and ≥65), a strong negative effect of donors ≥65 at 1 and 3 years posttransplantation, which lost significance when measured at 5 years posttransplantation, was found in their multivariable logistic regression analysis. On the other hand, their univariate analysis showed no differences between groups in the incidence of bronchiolitis obliterans syndrome (BOS), the most common form of chronic lung allograft dysfunction and the main limitation associated with long-term patient survival following lung transplantation (Bittle et al., 2013). The effect of donor age upon the risk of developing BOS was studied in more detail by Hennessy et al. who, also using the OPTN database, examined the association between different donor factors and the onset of this condition in 6991 recipients with relevant follow-up data transplanted between 1987 and 2008. Stratifying the donors by age into <60 and ≥60 groups, these authors found transplants from older donors to be an independent risk factor for the development of BOS during the first 5 years posttransplantation (Hennessy et al., 2010).

Using smaller cohorts and stratifying recipients into two groups according to donor age, three other studies also examined the effect of donor age upon patient survival following lung transplantation. In their univariate analysis and using a cut-off age of 50 and 55 years, respectively, Fischer et al. and Shigemura et al. did not find a significant effect of donor age in patient survival measured at different points between 1 month and 5 years posttransplantation, while De Perrot et al. found only a minor tendency to increased mortality at 30 days and 10 years posttransplantation for transplants from donors aged 60 years or older (Fischer et al., 2005; Shigemura et al., 2014; De Perrot et al., 2007). Therefore, data relating to the effect of donor age upon lung transplant recipient survival is somewhat conflicting, although an effect of donor age upon transplant recipient and graft survival from larger studies is clear.

### 2.6 Cornea (penetrating keratoplasty)

In a prospective clinical trial, the Corneal Donor Study (CDS) examined the effect of donor age upon graft survival in 1090 patients undergoing penetrating keratoplasty (PK) in different centers of the United States between 2000 and 2002 for an endothelial condition considered to be at moderate risk for graft failure (defined in the study as a regraft or a cloudy cornea that was sufficiently opaque to compromise vision for a minimum of three consecutive months). At five years posttransplantation no significant effect of donor age on graft survival was found for all the age range studied when donor age was analyzed as a continuous variable. The stratification of the donors into several age groups revealed similar survival curves across all groups, and only a slightly higher survival curve in the youngest group (12–30 years) (Gal et al., 2008). At 10 years posttransplantation, this effect became more marked and a second inflection point appeared after the fifth year posttransplantation toward the end of the age range, where the survival rate for the oldest group (71–75) reached a minimum. These apparent differences were supported by the association between donor age and graft survival found in the analysis at 10 years posttransplantaion with donor age treated as a continuous variable (Mannis et al., 2013). In parallel to the corneal donor study and using the same cohort, the Specular Microscopy Ancillary Study (SMAS) evaluated the effect of donor age on corneal endothelial cell loss posttransplantation, in which the recipients were stratified into those with transplants from donors 12–65 years old and those with transplants from donors aged 66–75 years. Apart from a substantial decrease in endothelial cell density (ECD) over the time in both groups, the authors found a slight but significant positive association between donor age and endothelial cell loss at 5 and at 10 years posttransplantation independent of the level of ECD present at the moment of transplantation (Lass et al., 2008; Lass et al., 2013). This finding is consistent with the decrease in ECD with age found in the corneas of healthy individuals in other studies (Sanchis-Gimeno et al., 2005; Armitage et al., 2014).

Other groups have also studied the effect of donor age upon graft survival following penetrating keratoplasty (PK). Wakefield et al. analyzed 9415 cases of PK performed in the UK between 1999 and 2012 and, after stratification of donors by age into 0–60, 61–75 and 76–90 year old cohorts and adjusting for different potential confounding factors, no significant difference in the rates of endothelial failure at 5 years posttransplantation were found between recipients of transplants from different donor age groups (Wakefield et al., 2015). Using the Australian Corneal Graft Register (ACGR) database for the period 1985–1996, Williams et al. analyzed data from 7741 patients with transplants from donors aged from <1 year to >90 years. In their analysis, stratifying donors by age in 10 years intervals, the authors were unable to find any significant effect of donor age upon graft survival up to 10 years post operation (Williams et al., 1997). However, in the 2015 ACGR report, based upon the outcomes of transplants performed between 2000 and 2014, a significant effect of donor age on graft survival following penetrating keratoplasty was found. The multivariate analysis showed that graft survival started to decrease in transplants from donors aged over 60. Although the report does not specify exactly when the differences between transplants from younger and older donors start to become significant, the overall results indicate that graft survival rates were equivalent in all groups during the first year and the differences between graft survival in transplants from older and younger donors increase progressively as they are reported for 3, 6 and 9 years posttransplantation (Williams et al., 2015). As such, the data suggest some effects of donor age upon graft survival in cornea transplantation, but further studies are warranted in order to reach stronger conclusions.

## 3 Discussion

The data available from organ transplantation show that increasing donor age negatively affects patient and graft survival in all organs studied where these two outcomes were measured (no data for graft survival in heart transplantation are presented here), as well as the risk of some other postoperative complications. Older donor age was also associated with a higher risk of graft failure following cornea transplantation. As one would expect from the normal process of aging, this effect of donor age upon survival outcomes is in general progressive, in the sense that it starts at a certain donor age and increases in tandem with increasing donor age. An exception to this pattern is the augmented risk of graft failure in transplants from pediatric donors that the studies by Baldwin et al. (2013) and Laging et al. (2012) found in lung and kidney transplantation data, respectively. Apart from these results, some studies in kidney transplantation have reported a relatively positive effect in patient or graft survival outcomes derived from age matching between donors and recipient (Chavalitdhamrong et al., 2008; Waiser et al., 2000; Lee et al., 2014). However, the number of recipients receiving grafts from old donors in these studies was not high enough to provide solid evidence of this. Even though an effect in this sense cannot be excluded, the results of different large-scale studies in kidney and other organ transplants suggest that, at least in the case of adult to adult transplantation, increasing donor age is associated with worse outcomes in all recipient age groups (Keith et al., 2004; Meier-Kriesche et al., 2002; Kasiske and Snyder, 2002; Nagji et al., 2010).

It is notable that some studies do not find any effect of donor age upon short-term postoperative outcomes despite finding this effect in the longer term. This could be due to any effects of donor age upon short-term patient and graft survival outcomes being masked by the comparatively greater effect of acute rejection and complications derived from surgical trauma upon short-term patient and graft survival outcomes. Other studies, however, do report a negative effect of donor age upon short-term patient and graft survival outcomes. Given the particularity of this early stage and the variability of results between studies, we have focused our comparisons upon studies analyzing longer-term outcomes (i.e. 6 months or more following transplantation). From an aging perspective, long-term studies are also more appropriate to study organ deterioration during aging.

The studies included in this review show that for each organ type, more or less defined patterns can be seen with respect to the age of the donor at which its negative effect on patient and graft survival outcomes becomes noticeable. In the case of the liver, three of the studies measuring graft survival via multivariate analysis in the short-medium term found this age to be between the third and the fourth decade of life (Marino et al., 1995; Detre et al., 1995; Feng et al., 2006). The results found for patient survival are consistent with these findings despite the fact that the intervals of donor age used to stratify the donors by Burroughs et al. were too wide to define this age (Marino et al., 1995; Detre et al., 1995; Burroughs et al., 2006). The study by Hoofnagle et al. (1996), however, did not find any significant effect on graft survival in its univariate analysis until donors aged <50–59 years. This is substantially higher than the donor age at which negative effects on graft survival become significant found by the other studies for liver transplantation included here (Marino et al., 1995; Detre et al., 1995; Feng et al., 2006), but because this analysis did not take into account possible confounding factors, this finding is less conclusive. It must be noted that the multivariate analysis showed that the poorer graft survival at 3 months posttransplantation conferred by older donors was restricted to those livers assessed by the harvesting surgeon as of poor or fair quality (Hoofnagle et al., 1996). It remains unknown, however, if this association would have been maintained in the longer term and also the effect that this variable would have had in the results of the other liver transplant studies reviewed if they had included it in their analyses.

The results observed for kidney are more homogeneous than those for liver. As seen in Table 1, four independent results (three using deceased donors and one using living donors) in three different studies found that donor age begins to have a significant negative effect on patient and graft survival at around 30–40 years (Laging et al., 2012; Keith et al., 2004; Oppenheimer et al., 2004). The study by Laging et al. (2012) also found improved patient and graft survival outcomes when using transplants obtained from living rather than deceased donors, a result reported in other studies and that, according to a study by Roodnat et al. (2003), could be explained in part, but not exclusively, by the longer periods of cold ischemia time used with deceased donors. Two additional studies comparing only two donor age groups confirmed the negative effect of donor age upon patient and graft survival, which was noticeable even when comparing donors 60–69 with donors >70 (Chavalitdhamrong et al., 2008; Moreso et al., 1999).

**Table 1:** Transplant studies investigating the effect of donor age. Unless otherwise stated, all the results presented are statistically significant.

Reference	Transplant type	Transplant year and country	Number of recipients	Outcome measured: type and timing	Recipient groups by donor age or continuous variable^f^	Donor age effect start	Confounders addressed?
Marino et al. (1995)	Liver	1992–1993 USA	462	Overall graft failure (f/u: 1.12 to 2.6 yr)	Continuous (range: 7–79^a^) [AI: <60 (408); ≥60 (54)]	45	Yes
Hoofnagle et al. (1996)	Liver	1990–1994 USA	772	Graft survival (at 3, 6, 12 and 24 mo)	6–20 (190); 20–29 (142); 30–39 (124); 40–49 (123); 50–59 (137); 60–73 (56)	50–59	No
				Graft survival (at 3 mo)	6–50 (579); 50–73 (193)	Older worse	Yes
Detre et al. (1995)	Liver	1987–1992 USA	7988	Overall graft failure (at 6 mo)	<20 (2299); 20–29 (2234); 30–39 (1489); 40–49 (1160); 50–76 (806)	30–39	Yes
				Patient survival (at 6 mo)		40–49 (no further progression)	
Feng et al. (2006)	Liver	1998–2002 USA	20,023	Overall graft failure (f/u: ≥1 yr; median = 3 yr)	0–39 (10,246); 40–49 (3752); 50–59 (3273); 60–69 (1896); >70 (856)	40–49	Yes
Burroughs et al. (2006)	Liver	1988–2003 Europe^d^	34,664	Risk of Mortality (at 3 mo)	<10–40 (11,241); 41–60 (7873); 60–>70 (2491)	41–60	Yes
				Risk of mortality (at 12 mo)	<10–40 (10,079); 41–60 (6942); 60–>70 (2033)		
Laging et al. (2012)	Kidney (DD)	1990–2009 Netherlands	941 (DD)	Death-censored graft failure and overall graft failure (f/u: 3 to 22 yr)	Continuous (range: DD = 0–79; LD = 15–80)	Above 30	Yes
	Kidney (LD)		880 (LD)			Above 30	
Keith et al. (2004)	Kidney	1990–1997 USA	50,322	Patient survival (at 5 and 10 yr)	<1–20; 21–25; 26–30; 31–35; 36–40; 41–45; 46–50; 51–55; 55–83 [AI: 0–6 (2141); 7–17 (8389); 18–29 (12,642); 30–41 (9729); 42–54 (10,761); >55 (6658)]	36–40	Yes
Oppenheimer et al. (2004)	Kidney (DD, LD)	1990–1998 Spain	3365^b^	Patient survival and graft survival (f/u: up to 10 yr)	<20; 20–30; 30–40; 40–50; 50–60; 60–70; >70 [AI: <60 (2887); >60 (478)]	30–40	Yes
Moreso et al. (1999)	Kidney	1984–1995 Spain	595	Overall graft failure (f/u: 1 to 12 yr)	<50 (485); >50 (110)	Older worse	Yes
Chavalitdhamrong et al. (2008)	Kidney	2000–2005 USA	9580	Overall graft failure, death-censored graft failure and patient death (f/u: up to 7 yr)	50–69 (8979); ≥70 (601)	Older worse	Yes
					60–69 (n/a); ≥70 (601)	Older worse (death-censored graft failure: NS)	
Gruessner and Sutherland (2005)	PTA	2000–2004 USA	453	Overall pancreas graft survival (at 1 and 3 yr) and risk of TF	<50 (440^a^); ≥50 (13^a^)	Older worse	Graft survival: No Risk of TF: Yes
	SPK		3947		<50 (3829^a^); ≥50 (118^a^)		
Gruessner (2011)	PTA	2006–2010 USA	450^a^	Overall pancreas graft failure (f/u: up to 5 yr)	0–14 (31^a^), 15–29 (n/a), 30–44 (n/a); >44 (27^a^)	NS	Yes
	SPK		4793^a^		0–14 (336^a^); 15–29 (n/a); 30–44 (n/a); >44 (288^a^)	30–44	
Sutherland et al. (2001)	PTA	1994–2000 USA	65	Overall pancreas graft survival (at 1 yr) and TF rates	PTA: <45 (56); ≥45 (9) SPK: <45 (141); ≥45 (33)	NS	No
	SPK		174				
Kayler et al. (2013)	SPK	1993–2008 USA	4636	Death-censored pancreas graft survival (at 3 yr)	<18–40 (3972); 40–>50 (664)	Older worse	Yes
Salvalaggio et al. (2007)	SPK	1994–2005 USA	8850	Death-censored and overall pancreas graft survivals (at 5 yr) and early graft loss (at 90 days)	1–45 (8074); 45–74 (776)	Older worse	Yes
Schenker et al. (2008)	SPK PAK PTA	1994–2006 Germany	340^e^	Pancreas graft survival (at 1, 5, and 10 yr)	<45 (271); ≥45 (69)	Older worse (due to TF)	No
Finger et al. (2013)	SPK (306) PTA (321) PAK (488)	1998–2011 USA	1115	Risk of TF (at 90 days)	n/a	>50 worse	Yes
Kutschmann et al. (2014)	Heart	2006–2008 Germany	774	Patient survival. (f/u: 3 yr)	Continuous (range: n/a) [AI: Interquartile range = 33.0–51.0]	Older worse	Yes
Stehlik et al. (2012)	Heart	1991–2010 Worldwide	7788 to 10,888	Patient survival (at 1, 5 and 15 yr) and risk of CAV (within 5 yr)	Continuous (range for period 2006–2011: <10 to >60)	Older worse	Yes
Weber et al. (2014)	Heart	2000–2012 USA	22,960	Patient survival (f/u: 5 yr)	18–39 (15614); 40–49 (4951); 50–54 (1491); 55–73 (904)	40–49	Yes
Gupta et al. (2004)	Heart	1990–2002 USA	667	Patient survival (f/u: mean = 4.5 yr)	Cut-points: ≥15, ≥20, ≥25, ≥30, etc. (range: 4–68)	40	Yes
Roig et al. (2015)	Heart	1998–2010 Spain	2102	Patient survival (f/u: up to 10 yr) and risk of CAV (at 1, 5 and 10 yr)	<50 (1758); 50–>60 (344)	Patient survival: NS CAV: Older worse at 5 and 10 years (not at 1 yr)	Yes
Nagji et al. (2010)	Heart	1987–2008 USA	39,704	Risk of CAV (f/u: up to 10 yr)	0–19.9 (9681); 20–29.9 (11614); 30–39.9 (8231); 40–49.9 (6966); 50–79.9 (3212)	30–39.9	Yes
Baldwin et al. (2013)	Lung	2005–2011 USA	8860	Overall graft failure (at 1 and 5 yr)	Continuous (range 6–76)	At 1 year: Likely over 50^c^	Yes
					6–18 (937);18–29 (3218); 30–54 (3837); 55–64 (769); 65–76 (99)	At 1 yr: ≥65 At 5 yr: NS	
Bittle et al. (2013)	Lung	2000–2009 USA	10,666	Patient survival (at 30 days and at 1, 3, and 5 yr)	18–34 (5367); 35–54 (4281); 55–64 (914); ≥65 (104)	At 1 and 3 yr: ≥65 At 5 yr: NS. At 30 days: NS	Yes
De Perrot et al. (2007)	Lung	1994–2005 Canada	467	Patient survival (at 30 days and at 10 yr)	9–60 (407); 60–67 (60)	NS	At 10 yr: No At 30 days: Yes
Fischer et al. (2005)	Lung	1998–2003 Germany	293	Patient survival (at 1 day and at 3, 6, 24, and 60 mo)	7–50 (244); 50–64 (49)	NS	No
Shigemura et al. (2014)	Lung	2003–2009 USA	593	Patient survival (at 30 and 90 days and at 3 and 5 yr)	<55 (506); ≥55 (87)	NS	Yes
Gal et al. (2008)	Cornea (PK)	2000–2002 USA	1090	Graft failure (at 5 yr)	Continuous (range: 12–75)	NS	No
					12–30 (69); 31–40 (58); 41–45 (63); 46–50 (68); 51–55 (126); 56–60 (152); 61–65 (171); 66–70 (217); 71–75 (166)		
Mannis et al. (2013)	Cornea (PK)	2000–2002 USA	1090	Graft survival (at 10 yr)	Continuous (range: 12–75)	Older worse	No
					12–30 (69); 31–40 (58); 41–45 (63); 46–50 (68); 51–55 (126); 56–60 (152); 61–65 (171); 66–70 (217); 71–75 (166)	Two possible inflection points^c^: 31–40 and 71–75	
Wakefield et al. (2015)	Cornea (PK)	1999–2012 UK	9415	Corneal endothelial failure (at 5 yr)	0–60 (3106); 61–75 (3329); 76–90 (2249)	NS	Yes
Williams et al. (1997)	Cornea (PK)	1985–1995 Australia	7741	Graft survival (at 10 yr)	0–10 (74); 11–20 (339); 21–30 (400); 31–40 (353); 41–50 (698); 51–60 (1204); 61–70 (1985); 71–80 (1933); 81–90 (435); >91 (20)	NS	No
Williams et al. (2015)	Cornea (PK)	2000–2014 Australia	8301	Graft survival (f/u: up to 12 yr)	0–19 (281); 20–29 (366); 30–39 (445); 40–49 (909); 50–59 (1578); 60–69 (1942); 70–79 (1974); 80–99 (786)	Over 60	Yes

DD = deceased donors; LD = living donors (unless otherwise specified, the donors used are DD).

TF: Technical failure.

PTA: Pancreas Transplantation Alone; SPK: Simultaneous pancreas-kidney transplantation; PAK: Pancreas after kidney transplantation.

PK: Penetrating keratoplasty.

NS: Not significant.

n/a: Data not available.

AI: Additional information.

f/u: Follow-up time.

yr: Year(s).

mo: Month(s).

a Approximate number of recipients estimated based on data provided in the article.

b Recipients with a functioning graft after the first year posttransplantation.

c Significance unknown.

d Twenty three European countries.

e Number of recipients of SPK, PAK and PTA: 317, 18 and 5, respectively.

f In brackets the number of recipient in each age group (categorical variables) or the total age range (continuous variables).

For pancreas transplantation, the only study that stratified donors into several age groups found those aged 30–44 to be the first that exerted a negative effect in graft survival (Gruessner, 2011). In addition, from the other studies it is apparent that when stratifying donors into two groups with a cut-off age of between 40 and 50 years, recipients of transplants from older donors have a higher risk of early graft loss due to technical failure (Gruessner and Sutherland, 2005; Schenker et al., 2008; Finger et al., 2013), a risk that in the study by Salvalaggio et al. (2007) is noticeable in medium to long term graft survival as well. These results have to be considered with caution because they are mainly obtained using data from SPK transplantation, a type of transplantation in which patients have also received a new kidney. This introduces a confounding variable into these results because SPK transplantation graft survival curves are known to be substantially higher than those observed in PTA. While one study measuring graft survival in PTA independently showed a similar result to that in SPK, additional studies in PTA patient and graft survival would be necessary to obtain any confident determination as to whether donor age exerts a similar relative effect in both types, as well as to arrive at a confident determination of how donor age affects pancreatic graft survival.

In heart transplantation, two studies suggest that medium-long term patient survival starts to significantly decrease in recipients of transplants from donors aged over 40 (Weber et al., 2014; Gupta et al., 2004), which is consistent with studies measuring the threshold for onset of CAV (30–39 years) (Nagji et al., 2010). The rest of the studies we reviewed confirm the negative effect of donor age in patient survival found by the above two analyses, except for that of Roig et al. (2015), in which the increased risk of death associated with the use of transplants from older donors (i.e. <50 vs ≥50 year old donors) found in the multivariate analysis lost significance after adjusting for donor cause of death, donor smoking history, recipient age, and induction and cyclosporine therapy. This contrasts with the results obtained by Kutschmann et al. (2014), where, of those potential confounders, only donor cause of death and recipient age were found to have an effect on patient survival, and donor age exerted a negative effect in patient survival that was independent of the age of the recipient. Also, it is remarkable that Roig et al. found a significant negative effect of donor age on the risk of developing CAV, which is a cause of mortality itself.

Two large scale studies show that donors over 65 strongly affect medium term patient and graft survival outcomes, respectively, in the context of lung transplantation, an effect that for graft survival starts to be noticeable from donors over 50 (Baldwin et al., 2013; Bittle et al., 2013). The lack of significance found in the rest of studies we reviewed for lung transplants, which used smaller cohorts and as such obtained less conclusive results, could also be consistent with these findings (Fischer et al., 2005; Shigemura et al., 2014; De Perrot et al., 2007).

Cornea transplantation outcomes appear to be much less affected by the age of the donor. The data reviewed here show that, with the possible exception of the 2015 ACGR report (Williams et al., 2015), none of the studies found a significant effect of donor age upon graft survival five years after PK, with donor ages ranging from <10 to >90 (Gal et al., 2008; Mannis et al., 2013; Wakefield et al., 2015; Williams et al., 1997). More evidence exists in the long term regarding the effect of donor age in graft survival. This lower effect of the age of the donor upon graft survival seems reasonable given the relatively simple structure of the cornea with respect to other commonly-transplanted organs and its lack of vasculature. The vascular system undergoes structural and functional changes with age that impair its function at different levels (Scioli et al., 2014; Ungvari et al., 2010), and as such it is logical to think that age-related changes in vasculature and microvasculature of the different organs must be a significant factor contributing to their age related dysfunction. The lack of vasculature of the cornea is also in part responsible for its known immune privilege, which makes the corneal grafts much less prone to rejection than other organs used for transplantation. In fact, in contrast to the necessary use of systemic immunosuppressants in recipients of any of the vascularized organs, topical administration of corticosteroids constitutes the immunosuppressive treatment for the patients of corneal transplantation (Niederkorn and Larkin, 2010).

In addition to patient and graft survival, some authors have studied the effect of donor age on the particular forms of chronic allograft dysfunction (CAD) in kidney, heart and lung transplantation (that is, CAN, CAV and BOS respectively), showing that older donor age is a risk factor for the development of each of these conditions (Moreso et al., 1999; Meier-Kriesche et al., 2002; Stehlik et al., 2012; Roig et al., 2015; Nagji et al., 2010; Hennessy et al., 2010). CAD, sometimes also referred to as chronic rejection, is a term used in solid organ transplantation to refer to a progressive decline of functionality of the transplanted organ that is accompanied by characteristic histopathological changes in it and that ultimately leads to graft failure since there is not an effective treatment for it at the moment. In heart and kidney transplantation, the effect of donor age upon CAV and CAN is clearly seen, along with consistently high incidences of these two conditions in the recipients, which suggests that the effect of donor age upon medium and long term graft and patient survival seen in heart and kidney transplantation could be due in a substantial part to its effect on CAV and CAN respectively. In lung transplantation, the similar incidence of BOS in different age groups found by Bittle et al., as well as the lack of significant effect seen by these authors and by Baldwin et al. of donor age upon patient and graft survival at 5 years posttransplantation, where the deaths due to BOS have been estimated to be >25% of all cases (Yusen et al., 2015), suggest that the effect of donor age upon the risk of developing BOS is more moderate. Liver and pancreas transplantation patients can also present CAD, but in these cases less is known about the extent of the effect of donor age.

Although each organ type presents a particular set of histopathological manifestations associated with CAD, there are some common features, such as interstitial fibrosis and fibrointimal hyperplasia of arteries, that affect all of them to some degree (Demetris et al., 1998). Etiologically, alloimmune-mediated processes are known to be the main causative factor leading to CAD, but it is known that non-immunological factors, such as hypertension, hyperlipidemia or postischemic reperfusion injury, can also contribute to its development, probably at least in part through different insults to the allograft that further exacerbate the innate immune response (Land, 2013).

Little is known about the mechanisms through which donor age influences the development of CAD. A possibility suggested by other authors in the case of heart transplantation is that older grafts could already have subclinical coronary disease at the time of transplantation, which could result in the earlier onset of CAV (Nagji et al., 2010). A more dysfunctional endothelium in the older graft could be a main causative factor because endothelial dysfunction, which is known to increase with age, leads to a more pro-inflammatory endothelium that favors atherosclerotic processes (Donato et al., 2015; Mudau et al., 2012). The unequivocal effect of donor age on CAD in heart and kidney transplantation, where the obliterative arteriopathy is of particular importance, compared to the less clear effect of donor age in this respect in lung transplantation, where the vascular disease is considered less relevant, strengthens this idea. Some studies with patients and animal models of kidney transplantation have also reported a stronger immune response in recipients with older donors during the early postoperative period which, through an elevated occurrence of acute rejections episodes, could increase the risk of CAD in the longer term (de Fijter et al., 2001; Reutzel-Selke et al., 2007).

### 3.1 Caveats and limitations

Although from the studies available in the scientific literature it seems evident that increasing donor age has a negative effect upon patient and graft survival outcomes in organ transplantation, attempting to obtain a more detailed picture of the extent of this effect in different organs is a challenging task. One limitation is the high variability to which this model is exposed. In addition to characteristics of donors with a potential confounding effect (e.g., cigarette usage, history of diabetes, cause of death and body mass index), intraoperative characteristics affecting grafts from young and old donors in distinct manners or bias in the assignment to recipients of these organs can distort the perceived effect of donor age. Hoofnagle et al. (1996), for example, found that the duration of the operation for the procurement of livers in older donors was significantly higher than in young donors. Also, some studies report a tendency to assign older grafts to older recipients, which would overestimate the negative effect of donor age in those studies and be a source of variability between studies. The identification of possible confounders and bias is complicated, especially given the retrospective approach used in most of the studies reviewed here.

It must be taken into account that the donors and their organs are subjected to a specific selection process and it is expected that the proportion of organs that are not accepted for transplantation will be higher among older donors. For example, the Corneal Donor Study did not use corneas with an endothelial cell density lower than 2300 cells/mm^2^, despite the fact that ECD decreases with age (Gal et al., 2008). A study by Potapov et al. also reported the use of <10% of the hearts from donors older than 63 years of age compared to a 60% utilization rate in younger donors (Potapov et al., 1999). Consequently, the dysfunctional status of the organs transplanted will not mirror that of the general population, with the proportion of grafts from older donors underestimating the real level of deterioration of that age group. This can be an advantage for a model trying to infer information about the aging rate or the functional deterioration in healthy subjects with age, as it would exclude those organs that are in worse condition, leaving a more healthy and homogeneous population of grafts in which more subtle differences could express themselves. However, this selection process can be an important source of variability between studies. Apart from being one of the main acceptance criteria itself, the age of the donor impacts other criteria used to assess the validity of the organs for use in transplantation and, if this quality control process is relatively more restrictive for some organs than for others, the respective outcomes would be less comparable. The same can be applicable to different studies for the same organ type, since the acceptance criteria in this respect can also vary between different countries and centers. For example, in 2009 in the United States 11.4% of all donors were 60 to 70 years old and 4.4% were older than 70, while the respective percentages for the same year in Spain were 19.5% and 25.4% (Halldorson and Roberts, 2013).

In addition, the relative impact that donor age has upon patient and graft survival can be influenced by the particular complications associated with each organ type, making the results from different organs less comparable for holistic analyses across multiple different organ types. For example, pancreas transplantation is known to have high rates of graft failure in the early stages following transplantation due to technical complications while, in the medium and long term, the incidence and clinical significance of CAD varies substantially between different organs, with patients of lung transplantation being the most affected in this respect, with rates of BOS approaching 50 and 75% at 5 and 10 years posttransplantation, respectively (Yusen et al., 2015). By contrast, several studies in liver transplantation have reported rates of chronic rejection as low as 3% (Jain et al., 2001; Jacob et al., 2005; Manousou et al., 2009), and non-hepatic complications derived from the immunosuppressant therapy, such as malignancies and infections, appear to be the leading causes of late mortality in these patients (Adam et al., 2012). These differences in the incidence of chronic rejection are in part a consequence of the differential levels of immunogenicity exerted by the different allografts, something that can also be seen in their propensity to achieve operational tolerance (i.e. spontaneous graft acceptance following cessation of immunosuppression). In adult liver transplantation, the rates of operational tolerance achieved after withdrawal from immunosuppression have been estimated to be between 8% and 33%. While rare, spontaneous operational tolerance is known to occur in patients of kidney transplantation as well, but in heart, lung and pancreas transplantation, organs that are considered to be more immunogenic, reports in this sense are almost non-existent (Madariaga et al., 2015; Chandrasekharan et al., 2013).

The results we review here seem to indicate a relatively low sensitivity of patient and graft survival to donor age in lung transplantation with respect to other abdominal and thoracic organs. This result begs the question of whether this is due to a real difference in the functional status of the organs transplanted or due to other factors. It is known that the outcomes of lung transplantation are particularly poor, only comparable to those of pancreas after PTA. For example, for adult patients receiving an organ from a brain-dead donor in the UK between 2003 and 2005, 5-year patient survival rates for liver, heart and lung were 77%, 67% and 52% respectively, while the 5-year graft survival for kidney, pancreas after SPK and pancreas after PTA were 84%, 72%, 50% respectively (Johnson et al., 2014). One possibility is that in a situation of higher pressure toward graft failure and death, the effect of donor age would need to be stronger in order to noticeably affect patient and graft survival outcomes.

Apart from the limitations discussed above, an additional obstacle that this review faces is the variability between studies in key aspects such as the outcome measured (overall graft survival, death censored graft survival and patient survival), follow-up time (short-, medium- and long-term analyses), year of transplantation (which fails to take into account the continuous improvements of postoperative outcomes seen over the years as a consequence of advances in transplant surgical techniques, organ preservation, postoperative care and immunosuppressive therapy) and other confounding factors that differ (in terms of the number and type of confounding factors analyzed) from procedure to procedure and from analysis to analysis. Other particularities of the studies presented here also limit the potential of this systematic review to infer information about organ and organismal aging. Only a few studies took into account the age of the recipients together with the age of the donors in their analysis, therefore lacking results on the effect of donor and recipient age in the different donor-recipient age combinations. Likewise, the inclusion of more posttransplantational data informing of the physiological or histological status of the transplanted organs over time or the use of longer follow-up times in some of the studies would have enhanced their value from an aging research perspective.

Despite the numerous limitations and difficulties of our approach, with the currently available and continuously increasing vast amount of data from organ transplantation, more tailored experimental designs could be implemented in order to minimize or overcome some of these limitations and exploit the big potential that these data have in aging research. Some limitations, however, seem particularly difficult to solve even with more tailored experiments. For example, in our attempt to infer differential aging rates between organ types based on the effect of the age of the donor in the outcomes, we find that the particular set of posttransplantation complications and course of the outcomes associate with each organ type represents an unsolvable obstacle for this endeavor at the moment.

### 3.2 Conclusion & future research directions

The results from organ transplantation possibly reflect the functional decline that different tissues and organs undergo with age. Despite the limitations inherent in reviewing results from studies that analyze patient and graft survival outcomes in transplants and the particular limitations that the studies included in this review may have (see Section 3.1), the results presented here show that for each organ type, patterns are found with respect to the age of the donor at which its negative effect upon patient and graft survival outcomes becomes significant. However, knowing how much this observed threshold correlates with the dysfunctional status or the aging in each organ type enough to make comparisons between them is a more complex approach that is unlikely to succeed given the particular complications of each organ transplantation type.

At present, the data from human organ transplantation represents a unique and valuable resource in biogerontology that with appropriate experimental design could help us to gain a better understanding of some of the processes involved in organ and organismal aging. Breakthrough advances will be needed, however, to overcome the long term immunological consequences of receiving an allograft, either in the form of chronic rejections or as complications derived from generally lifelong immunosuppressant therapy, which together are the main limitations to long term survival and currently an unavoidable source of variability between organs types. In this sense, new strategies for the induction of tolerance, which are already showing positive results in liver and kidney human transplantations and limited success in heart and lung transplants in animal models (Kawai et al., 2014), have the potential to significantly reduce this obstacle, increasing the survival rates of grafts and patients and normalizing the course of patient outcomes between the different organ transplantation types, thereby creating a more appropriate landscape for their comparison.

The successful implementation of therapies for the induction of tolerance in the different organs would also lead to cases of organs with a total age that exceeds that of the known natural limits of human longevity, especially if the reuse of transplanted grafts becomes a more regular practice. Without having to wait for this optimistic scenario to arrive, the data currently available allows predicting that, among the vascularized organs, some cases could eventually appear in the current transplantation era. Probably one of the most extreme cases reported to date is that of a liver from a 93 years old donor transplanted into a 19 year old recipient, who seven years later was still alive and without major complications (Tolan et al., 2016). In corneal transplantation, the more extended use of transplants from very old donors and the superior outcomes made in this arena suggest that this limit is already being approached. Among the records of the Danish Cornea Bank, Visby et al. (2014) identified 88 recipients with corneas with a total age of between 100 and 118 years, with the oldest one still functioning by the time of their study in a 57-year-old woman.

Finally, in animal models, the possibility of performing consecutive transplantations of organs between syngeneic individuals offers the possibility of following the aging of individual organs without the immunological derived problems and without the limitations imposed by the lifespan of the organism. Harrison (1973) performed serial transplantation of bone marrow across successive generations of mice, executing a total of four serial transplants across 36 months, and demonstrated that the bone marrow remained functional throughout its serial transplantation, and most notably, retained its functionality past the maximum lifespan of its initial donor (Harrison, 1973). Lee et al. serially transplanted pancreaticoduodenal and single kidney grafts between syngeneic Lewis rats, obtaining pancreaticoduodenal grafts of up to 42 month and kidney grafts of up to 32 months after, respectively, 5 and 3 rounds of consecutive old to young transplantation, which is substantially higher than the average life expectancy of 24 months of these animals (Lee et al., 1997, Lee et al., 1999). These results may be explained by systemic factors. Indeed, heterochronic parabiosis studies have revealed a number of rejuvenating effects resulting from heterochronic parabiosis in murine model organisms (Conboy et al., 2013). Together with evidence of hypothalamic programming of systemic aging (Zhang et al., 2013), these results show that improvements of organs transplanted from the old to the young can occur due to systemic factors, and that the abovementioned possibility of transplanted organs surpassing human longevity is plausible.

In conclusion, using data from organ transplants is a largely unexplored approach in aging research that could provide a powerful platform for deriving novel biogerontological data and insights.

## Declaration of interest

None.
